# Looking Across and Looking Beyond the Knowledge Frontier: Intellectual Distance, Novelty, and Resource Allocation in Science

**DOI:** 10.1287/mnsc.2015.2285

**Published:** 2016-01-08

**Authors:** Kevin J. Boudreau, Eva C. Guinan, Karim R. Lakhani, Christoph Riedl

**Affiliations:** London Business School, London NW1 4SA, United Kingdom; and Harvard Business School, Boston, Massachusetts 02163; Dana-Farber/Harvard Cancer Center, Boston, Massachusetts 02215; Harvard Business School, Boston, Massachusetts 02163; D’Amore-McKim School of Business, Northeastern University, Boston, Massachusetts 02115

**Keywords:** innovation, project selection, bounded rationality, knowledge frontier, novelty, intellectual distance

## Abstract

Selecting among alternative projects is a core management task in all innovating organizations. In this paper, we focus on the evaluation of frontier scientific research projects. We argue that the “intellectual distance” between the knowledge embodied in research proposals and an evaluator’s own expertise systematically relates to the evaluations given. To estimate relationships, we designed and executed a grant proposal process at a leading research university in which we randomized the assignment of evaluators and proposals to generate 2,130 evaluator–proposal pairs. We find that evaluators systematically give lower scores to research proposals that are closer to their own areas of expertise and to those that are highly novel. The patterns are consistent with biases associated with boundedly rational evaluation of new ideas. The patterns are inconsistent with intellectual distance simply contributing “noise” or being associated with private interests of evaluators. We discuss implications for policy, managerial intervention, and allocation of resources in the ongoing accumulation of scientific knowledge.

## 1. Introduction

A fundamental challenge that all organizations engaged in scientific and technological innovation face is how to allocate resources across alternative project proposals (e.g., Astebro and Elhedhli 2006, Hallen 2008). Senior managers and scientific researchers alike devote significant time and effort to evaluating and selecting projects. Stevens and Burley (1997) find that executives have to manage, on average, more than 3,000 ideas to secure one commercial success. In science, tens of thousands of experts are involved in the annual evaluation of more than 89,000 research applications by the National Institutes of Health (NIH) and National Science Foundation (NSF) (Stephan 2012, Li 2015). The challenge of evaluating ideas has only grown with increasing use of “ideation,” platform-based contests, crowdsourcing, and crowdfunding as a means of generating a large number of proposals (Agrawal et al. 2014, Piezunka and Dahlander 2014, Mollick and Nanda 2015). A common approach to evaluating innovative projects is to refer to experts with deep domain knowledge to assess quality of proposed projects, i.e., peer review (Chubin and Hackett 1990, Lamont 2009). In the United States, for example, academic research, which is the feedstock for many subsequent commercial innovations, depends on expert peer review to allocate more than $40 billion of research funds every year in engineering, medicine, science, and technology (Xie and Killewald 2012). Contrary to the popular notion of a “marketplace for ideas,” in which the best ideas simply rise to the top, resource allocation in academic science is shaped in important ways by supporting institutions and processes (Kuhn 1962, Merton 1968, Dasgupta and David 1994, Stephan 2012). In this paper, we investigate how “intellectual distance”—the degree of overlap and relatedness between evaluators’ knowledge or expertise and the knowledge embodied in research proposals—plays a role in systematically shaping evaluation outcomes and consequent resource allocation in scientific peer review.

The evaluation and funding process for leading-edge scientific and technological projects is highly competitive. In the United States, for example, the NIH funds fewer than one in six applications, and for the NSF, it is one in four. Between one-third and one-half of rejected project proposals and their associated research lines are subsequently discontinued by their authors (Chubin and Hackett 1990). Although rejected proposals might simply be of lower quality and deserve to be stopped, tremendous unexplained variation and seeming “noise” is the single most regular feature of scientific peer evaluations. Interrater reliability in funding decisions is routinely found to be very low (e.g., Rothwell and Martyn 2000, Bornmann and Daniel 2008, Jackson et al. 2011), with concordance sometimes “barely beyond chance” (Kravitz et al. 2010, p. 1) and “perilously close to rates found for Rorschach inkblot tests” (Lee 2012, p. 862). Variance among reviewers is sometimes greater than variance between submissions (Cole et al. 1981). Beyond the fact of low interrater reliability, there is yet little agreement about underlying causes. Past research has argued that expert evaluation of research proposals may be shaped by any number of factors beyond the “true” quality of research, including researcher and evaluator characteristics, ties between researchers and their evaluators, proposal formats, and evaluation procedures. (See Marsh et al. 2008 and Lee et al. 2013 for comprehensive reviews and syntheses of the relevant findings.)

In this paper, we investigate whether the intellectual distance and relative positions in “knowledge space” between evaluators’ knowledge and the knowledge embodied in research proposals has systematic effects on evaluations. We motivate and study two specific conceptions of distance: intellectual distance per se and novelty, or departures from the established body of research.

We consider three theoretical perspectives and associated mechanisms through which positions in knowledge space might affect evaluations, independent of the true quality of a proposal. First, the evaluation process might simply be understood as a matter of evaluators each discerning a noisy signal of true quality, following a classical statistical *decision making under uncertainty* perspective. In this case, greater intellectual distance (less expertise, greater ignorance) would lead to less precise evaluations but no differences in mean evaluations. By contrast, a *bounded rationality perspective* predicts that cognitive limits and closely associated behavioral and heuristic responses lead to systematic biases with intellectual distance. An *agency perspective* suggests the possibility that some evaluators might adjust their evaluations—one way or another—in response to private interests.

Our empirical task is to precisely observe variation in intellectual distance and relate this to evaluation outcomes, independent of conflating factors, including the true quality of research proposals. A deep and fundamental challenge for research of this kind is that the true quality and potential of a research proposal is not observed and difficult to unequivocally infer–even after, if and when, the research is finally executed (e.g., Merton 1968). Therefore, a key feature of our research is to devise an approach to deriving inferences that does not rely on observing true quality.^1^

To implement a suitable experimental research design, we collaborated with the administrators of a research-intensive U.S. medical school. We devised ways of modifying details of a research grant process for endocrine-related disease to allow us to make experimental comparisons. We then worked closely with the grant organization to manage, administer, and execute details. Key aspects of the design included recruiting an especially large number of evaluators, 142 world-class researchers from within the institution that were drawn from fields both inside and outside the disease domain. We randomly assigned each evaluator to 15 proposals from a total of 150 research proposals, yielding 2,130 evaluator–proposal pairs. The process was “triple blinded,” with evaluators and authors blinded to one another, and evaluators, too, blinded to one another. Focusing our analysis on the first stage of the grant process, in which ideas and new hypotheses were solicited and evaluated, allowed us to standardize the format and content of proposals and to simplify submission requirements so that we could restrict the process to single-author submissions. Thus, we could associate each proposal with fine-grained metrics at the level of individual submitters and evaluators.

We found that evaluators gave systematically lower scores to research proposals that were closer to their own areas of expertise. The relationships are strikingly large and driven by behaviors across a wide mainstream of the population. The variation in intellectual distance across this group of medical researchers accounts for 1.1 points of variation on a 10-point evaluation scale. (The standard deviation of evaluation scores overall is 1.7 points, after removing proposal and evaluator fixed effects.) Given the research design, these can be interpreted as causal effects. Simulating an alternative ranking scheme, we find that intellectually “closest” expert evaluators would have generated scores that would lead proposals to change their rank order by over 30 positions, on average. The evidence suggests that experts’ rank ordering is more meaningful than is that of averages of larger groups of (less expert) evaluators, particularly among highest-quality proposals.

Our second main finding is that more novel proposals are associated with lower evaluations, with magnitudes of effects comparable to those associated with intellectual distance. It is proposals with particularly high levels of novelty—the “right tail” of novelty—that account for this result. (For low levels of proposal novelty, evaluation scores were increasing with incrementally greater levels of novelty.) It is, of course, not possible to experimentally vary the novelty of a proposal entirely independently of its other characteristics. Therefore, it is important to note that we instead implemented a best feasible approach to estimate the relationship between novelty and evaluation scores, all else being equal. We use a series of specifications and diagnostics to rule out omitted variable bias.

These and each of a number of other patterns studied herein are consistent with biases rooted in bounded rationality in a context of especially high uncertainty (Kahneman et al. 1982, Johnson et al. 1982, Camerer and Johnson 1991). In relation to intellectual distance, the pattern of lower scores provided by most expert evaluators is consistent with experts more readily “seeing” and “sampling” more informational cues than do less expert evaluators—with experts observing a disproportionately greater number of demerits, problems, and limitations of research proposals. In relation to novelty, the pattern of lower scores associated with novel proposals, along with other patterns, is consistent with boundedly rational evaluators systematically misconstruing ideas outside the established paradigm.

The range of patterns in these data is inconsistent with characterizations of the evaluation process as simply one of inferring true quality from noisy signals, as in classical statistical decision making under uncertainty characterizations. Findings are also inconsistent with evaluators being biased by private interests. We are, however, unable to rule out the possibility that novelty is somehow inevitably and inextricably associated with truly lower mean expected outcomes.

These findings have profound implications for evaluation of frontier projects. First, these effects are insensitive to usual procedures such as blinding of the identity of researchers. Second, unlike, say, the evaluation of prices in markets or product ratings by online “crowds,” bounded rationality implies limits to what can be achieved by tallying and aggregating large numbers of opinions. Third, whereas problems with intellectual distance have the potential to “scramble” rank ordering, problems with novelty have the potential to systematically dissuade experimentation.

The remainder of the paper proceeds as follows. Section 2 reviews past literature and motivates possible links between intellectual distance and evaluations. Section 3 describes the research design. Section 4 presents main results. These are discussed and interpreted in §5, together with a series of supplementary discriminating tests. Section 6 concludes.

## 2. Advancing Scientific Knowledge and Evaluations

In this section, we first describe how recurrent patterns of knowledge accumulation in science inevitably lead to some degree of intellectual distance between new research proposals and the knowledge of evaluators. We distinguish intellectual distance between particular pairs of research proposals and evaluators from novelty in relation to the entire existing body of research. We then discuss three distinct theoretical perspectives suggesting intellectual distance might shape and evaluations, independent of the true quality of a proposal. (Note, each of the perspectives reflects vast literatures, and we only provide a brief overview of arguments as a means of summarizing key differences in their implications.)

### 2.1. Intellectual Distance and Novelty in the Advance of Scientific Knowledge

Advances in scientific knowledge tend not to be a scattershot of isolated experiments in all directions but rather a series of regular accumulative patterns (Gibbons et al. 1994). Initial progress on the resolution of a scientific problem gives rise to a scientific paradigm (Kuhn 1962), defined as common knowledge and consensus on what is to be observed, which questions are legitimate and interesting to ask, what constitutes appropriate and useful approaches to addressing these questions, what methods might be fruitfully employed, and even what legitimate answers might look like. Thus, except in the rare instances in which one paradigm is abandoned for another, the stock of knowledge tends to grow by regular accretion within the prevailing paradigm.

Disclosure and diffusion of scientific knowledge through publication, conferences, seminars, textbooks, graduate training, and other means creates something of a common stock of open knowledge (Boudreau and Lakhani 2015), as well as a commonly perceived knowledge frontier or an envelope that demarcates what is currently known from what remains to be investigated. New research, which by definition aims to extend the current state of knowledge, creates intellectual distance between evaluators and proposals if only by requiring evaluators to look *beyond* the existing knowledge frontier. Incrementally novel advances can be made by continuing within existing pathways and paradigms. More novel departures from the existing paradigm might also be pursued, in hopes of finding new viable research pathways and “breakthroughs” (Uzzi et al. 2013). Thus, novelty should be considered a matter of degree. Just as incremental advances largely proceed in a cumulative process that draws on existing templates, knowledge, and ideas, novel departures themselves do not come from utterly unprecedented work. Rather, as documented in a range of empirical and theoretical considerations (Weitzman 1998, Fleming 2001, Uzzi et al. 2013), novel approaches themselves draw on existing knowledge but tend to then recombine and reconfigure this knowledge in unprecedented ways.

**Figure 1:**
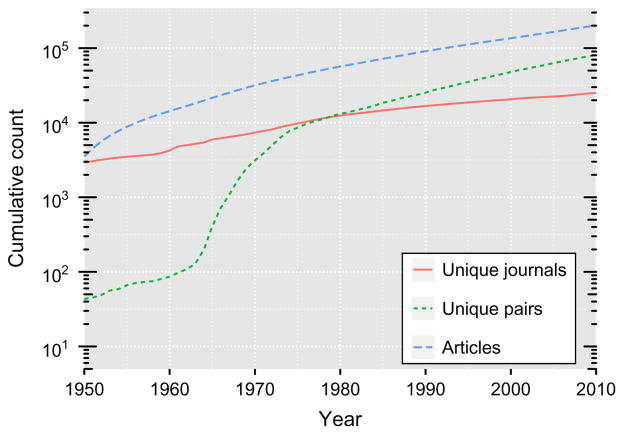
Time Trend of Cumulative Numbers of Publications, Unique Journals, and Unique Pairs of Keyword Topics and Article Counts *Notes.* Based on data from the PubMed database. Keywords are based on standardized lexicon (MeSH terms).

Intellectual distance between a particular evaluator and particular research proposal also arises as a result of growing specialization as scientific research advances. Despite the open and shared knowledge commons, scientific knowledge remains too vast, nuanced, and complex to be understood in its entirety by any one scientist (Cowan et al. 2000, Jones 2009). Figure 1 illustrates the growth of scientific knowledge in the life sciences over 60 years (1950–2010) and the tendency toward specialization into subfields through the increase in the cumulative numbers of journals, articles, and research keywords. Even scientists that prima facie appear to be working in the same domain will differ in the particulars of their research program and differ in precise experience, training, and exposure to phenomena and methods. As a result, evaluation of new research proposals also requires evaluators to look *across* the knowledge frontier to other domains not precisely overlapping with their own expertise, training, and experience. Hence, the very nature of scientific inquiry and our society’s reliance on experts to evaluate and allocate resources generates intellectual distance between evaluators and new proposals and creates evaluation challenges.

### 2.2. Three Perspectives on Intellectual Distance and the Evaluation of New Projects

Here, we review three broad theoretical perspectives, each motivating possible links between intellectual distance and research evaluations, apart from any differences in true research quality. Although these perspectives are not mutually exclusive or entirely independent, it is useful to consider their arguments in turn. Predictions of these perspectives are summarized in Table 1.

#### 2.2.1. Agency Problems and the Private Interests of Evaluators

Much of the existing research on research evaluations hypothesizes some form of evaluator bias shaping evaluations. Most existing evidence is correlational and associative and not yet directly related to the question of intellectual distance.^2^ Nonetheless, we take the more general point emphasized by this work that evaluators’ private interests might lead to systematic deviations between expected quality and reported evaluations. Even just the content of a research proposal may relate to private interests of evaluators. For example, a negative relationship between evaluations and intellectual distance could exist, if evaluators are inclined to be less critical of or to favor “close” research. This is plausible given the nature of institutions and rewards in science (Stephan 1996). Increased attention can attract additional resources and renown for one’s area of research, boosting the prospects of all involved—including the evaluator. Equally, a negative relationship could exist if evaluators’ have preferences for given “schools of thought” or have a propensity for “cognitive cronyism” (Travis and Collins 1991, p. 323). Alternatively, a positive relationship could exist where, for example, research in the same domain and in close proximity is perceived to exert a negative externality on the evaluator, creating incentives to discount evaluations. For example, in certain instances, a close and competitive proposal might be expected to draw resources and attention away from an evaluator’s own work (Campanario and Acedo 2007). Similarly, a wish to “protect” orthodox theories might dispose evaluators to look negatively at research that is both proximate and proposes a conflicting perspective (Travis and Collins 1991). These biases, in whichever direction, might also occur more subtly than simply evaluation in bad faith, as when personal interests affect how much effort an evaluator is willing to devote to an evaluation (Johnson and Payne 1985).

Past empirical research with some relevance to these arguments is not conclusive on these points. For example, several papers have failed to find upward bias in evaluations of research that cites evaluators (Sandström and Hallsten 2008, Sugimoto and Cronin 2013). Li (2015) finds clearer evidence of a positive causal bias toward close researchers in the context of NIH committee evaluation; however, committee dynamics and nonblinded evaluations make it difficult to interpret results in relation to intellectual distance per se.

#### 2.2.2. Uncertainty, Risk, and Decision Theory Perspectives

Another theoretical perspective views proposal evaluation as akin to the problem of classical (statistical) decision making under uncertainty (e.g., Berger 1985, Anand 1993). This might be understood in terms of reported evaluation scores (*V* ^reported^) being understood as reflecting both some true, unobserved quality (*V* ^true^) and some “error” term (e.g., Blackburn and Hakal 2006, p. 378); i.e., *V* ^evaluation^ = *V* ^true^ + *error*. This perspective is implicit in the many references to “luck” and “noise” in the literature (e.g., Cole et al. 1981, Marsh et al. 2008, Graves et al. 2011). This view also relates to the common practice of averaging multiple evaluation scores in hopes of canceling noise and errors (Lee et al. 2013).

Following this view, greater intellectual distance can be interpreted as an evaluator being less well informed—and therefore making an evaluation under greater uncertainty. Greater intellectual distance and uncertainty might perhaps manifest as a larger “error” term, potentially producing greater dispersion and variance among more distant evaluators without necessarily having any effect on mean evaluations. Alternatively, greater intellectual distance and uncertainty might reduce confidence in assessments—even where it has no effect on one’s evaluation—possibly encouraging discounting for perceived risk of more distant evaluations.

Novel research proposals may face an added hurdle. Apart from uncertainty in the form of risk or errors, novelty introduces a form of fundamental uncertainty that can not entirely be resolved without experimentation. It is thus difficult to assign probabilities to outcomes ex ante. In cases of such unresolvable uncertainty or “ambiguity,” researchers in the behavioral decision making under uncertainty literature have found that individuals tend to discount outcomes on the basis of “ambiguity aversion” (Fox and Tversky 1995). This reasoning also predicts a negative relationship between novelty and evaluations.

#### 2.2.3. Bounded Rationality and Expert Cognition Perspectives

Research on bounded rationality and expert cognition also suggests links between intellectual distance and evaluations. The literature in this tradition finds that, across a wide range of human endeavor, expert judgment is associated with qualitatively distinct cognitive processes from those of non-experts. Experts, those closest to a particular subject matter, are able to observe and exploit a far broader array of informational cues. They perceive and appreciate more detail, complexity, patterns, and meaning when making the very same observations as nonexperts (see Kahneman et al. 1982, Johnson et al. 1982, Camerer and Johnson 1991). These advantages in information processing are rooted in the development of a richer, more textured library of domain-specific knowledge accumulated through extended periods of training, experience, and practice. As a result, experts require the same or less time and effort to generate more discerning judgments (Johnson and Russo 1984, Johnson 1988, Bedard 1989). Expert cognitive processes are even often seemingly automatic, and even instantaneous, as a result of knowledge stored and comprehended in “chunks” and mental maps of hierarchies, relationships, contingencies, and “configural rules” (Fitts and Posner 1967, Newell and Simon 1972, Chase and Simon 1973, Ericsson and Smith 1991).

Therefore, rather than a matter of intellectual distance resulting in more or less “error” in perceiving the same object, these points raise the possibility of information processing and “seeing more” creating differential *sampling* of information. Following this interpretation, the effect of intellectual distance and expertise depends on whether experts disproportionately see (sample) merits or demerits in relation to those perceived (sampled) by less expert evaluators. It is only when experts differentially sample positive merits as they do negative demerits of a research proposal (and also weight them equally) where we would not expect some effect of expertise on mean evaluations. If merits and contributions are much plainer to see than are more subtle questions of feasibility, implementation, and correctness, greater expertise could result in more negative evaluations. This suggests the possibility of a positive relationship between intellectual distance and evaluations.

A distinct branch of the research on cognitive biases, studying effects of extrapolating on the basis of one’s existing knowledge into new domains, also suggests implications around questions of novelty. Extrapolation beyond the domain for which knowledge was developed has been documented to result in sharply degraded performance, even to the point that human judgment becomes inferior to naïve actuarial models (e.g., Johnson 1988, Sternberg 1996, Chi 2006). Expert mental maps have thus been described as “brittle” (Camerer and Johnson 1991) and subject to breakdown when applied to new areas (Brehmer 1980, Holland et al. 1986, Camerer and Johnson 1991, Chi 2006). These findings suggest that novel approaches might be systematically “misconstrued” if uncertainty surrounding them leads them to be interpreted on the basis of existing knowledge and mental maps. If this leads to discounted evaluations, a negative relationship between evaluations and “novel” research proposals will manifest.

### 2.3. Summary and Research Questions

Intellectual distance is a regular feature of the evaluation process and deserves careful study as a variable that might influence evaluation and resource allocation in science. The theoretical perspectives reviewed above and the mechanisms they suggest are summarized in Table 1, with predictions in relation to mean evaluations. Several points relate specifically to the case of novel departures from existing research approaches. Our main goal in this study is to test for systematic relationships between evaluation scores and intellectual distance. A secondary goal is to attempt to rule in and rule out alternative theories.

## 3. Research Design

In this section, we describe the setting and research design, providing details on proposal generation, evaluator recruitment, random assignment, and our key measures.

### 3.1. A Call for Research Proposals from the “First Phase” of a Grant Process

We carried out our research in the context of a scientific grant solicitation and evaluation process for research on endocrine-related disease, a major economic and health burden on society and a focus of considerable research effort at the host medical school. Working closely with grant administrators, we altered the usual grant procedures to allow us to make precise observations and to derive meaningful inferences. The grant process we studied involved seed grant awards, intended to enable investigators to initiate their research efforts to generate preliminary data (to support later NIH grant applications).

In terms of defining the scope, we deliberately defined the grant solicitation in terms of a disease area rather than making any mention of existing literature, the existing body of scientific knowledge, or established research pathways. The articulated aim for the grant was otherwise stated in general terms of directing research attention and financial resources to make progress in endocrine system–related disease research, treatment, and care. The content of proposals was otherwise unconstrained; we welcomed submissions related to diagnosis, treatment, and pro-phylaxis. To attempt to draw a variety of submissions, the university president communicated an open call to participate to all members of the medical school and broader university community via email.

A fundamental research design choice was to partition the grant proposal process into two phases. The first, involving solicitation of proposals for approaches and ideas, was essentially a call for research hypotheses. It is this first phase—of defining research goals, approaches, and hypotheses—that is most relevant to the questions raised earlier (in §2). Partitioning the proposal process in this manner also reduced “entry costs” to prospective submitters, making it possible to document submissions in shorter proposals. (The average proposal length in this exercise was roughly six pages.) This design decision also allowed us to require submissions be authored by *individual* scientists rather than teams. Thus, we could associate each proposal with the attributes of the individual submitter. The shorter and more standardized proposal format also allowed us to minimize the extent to which submission format shaped evaluations (Langfeldt 2006).

Explicit incentives in this process included a $2,500 cash prize awarded to each of the top 12 winners. The process also generated additional incentives: the winning proposals would form the basis for a call for research proposals, the second phase, in which a total of $1 million in seed grants would be available. Being in the top of the first phase increased the odds of being able to create a successful second-stage proposal. (Indeed, four second-phase winners were also first-phase winners.) The first phase of the process also served as a platform for high-profile exposure among peers and university leaders, as awards were conferred by the dean of the medical school in a formal public ceremony attended by colleagues, White House staff, and members of the media. This process elicited 150 research proposals, with 72 coming from within the host university.

### 3.2. Recruiting Evaluators

Major funding agencies regularly invite researchers with relevant subject knowledge to participate in evaluating research proposals (Langfeldt 2006). An ad hoc evaluation team might include a few, perhaps five to seven (Langfeldt 2006), specialized researchers whose phenomenological interest, research methods, and/or topical focus relate to the research proposal(s) in question (Jayasinghe et al. 2003). More extensive evaluation processes covering large numbers and steady flows of proposals, such as those employed by the NIH and NSF, often involve standing committees and subcommittees formed around topic areas to which proposals are directed, as appropriate. Such committees can be as large as 30 to 50 researchers (Li 2015) and their identities publicly disclosed.

Given our interest in generating variation, as well as abundant replication and degrees of freedom, we recruited roughly equal numbers of evaluators from among three distinct groups of host university faculty: (i) those with at least one publication in the disease area, (ii) those without publications in the particular disease area but with at least one publication with someone with a publication in the disease domain, and (iii) those without any publications or links to the disease area. Within each of these groups, we recruited equal numbers of senior and junior faculty (30 of each). We populated these six groups by rank-ordering faculty at the medical school according to publication counts and inviting the top-ranked faculty from each of the three groups to participate. Drawing on faculty from the host university ensured high-caliber participants, independent of rank. Strong institutional support helped minimize dropout. Of the 180 invitations (i.e., 6 groups times 30 invitations per group), 142 individuals accepted and participated in the exercise. This produced roughly equal proportions, balanced across the groups in relation to both the literature and junior and senior scholars. Each group also reflects considerable diversity in gender, age, and training (in terms of M.D. or Ph.D.). The group is uniform in including just highly accomplished researchers, with an average publication count of 101. Submitters are themselves accomplished but clearly more junior, on average, with roughly 1/10 as many publications, on average.

### 3.3. Evaluator Assignment and the Evaluation Process

Our assignment of evaluators and proposals yielded 2,130 proposal–evaluation pair observations. Ten blocks of 15 research proposals, randomly drawn from 150 total, were randomly assigned to each of the 142 evaluators, giving an average of 14.2 randomly selected faculty per proposal. Block randomization in this fashion was implemented to ease back-office implementation of the procedure by administrators at the institution.^3^ Following convention in medical research grant proposal evaluations, the task of evaluators was to score proposals by responding to the question, “On a scale of 1 to 10 (1 [being the] lowest to 10 [being the] highest) please assess the impact on disease care, patients, or research.”

Given our interest in having evaluators respond to the content of proposals rather than the identities of submitting researchers, we designed the process to minimize the probability of identities being revealed. Submitters’ names were blinded on proposals, and evaluators, whose identities were also blinded, performed their evaluations independently and had access only to the 15 assigned proposals. Evaluators neither knew the names of nor interacted with other evaluators. With evaluators thus effectively blinded from one another, the overall evaluation process was triple blinded.

### 3.4. Data Collection and Variables

Our central concerns are to measure the relationship between evaluation scores and intellectual distance and to novelty in relation to existing research. We therefore devised means of measuring these key objects and identified several control variables relevant to our analysis. The data set includes evaluators’ score sheets, submitted proposals, detailed backgrounds, and résumés (of those evaluators and submitters at the host university) from the host university’s database; third-party topical keyword coding of submissions; and the PubMed database (an extensive database of research publications in life sciences). An overview of definitions and summary statistics for the main variables are provided in Tables 2 and 3, respectively.

#### 3.4.1. Evaluation Scores

**Figure 2:**
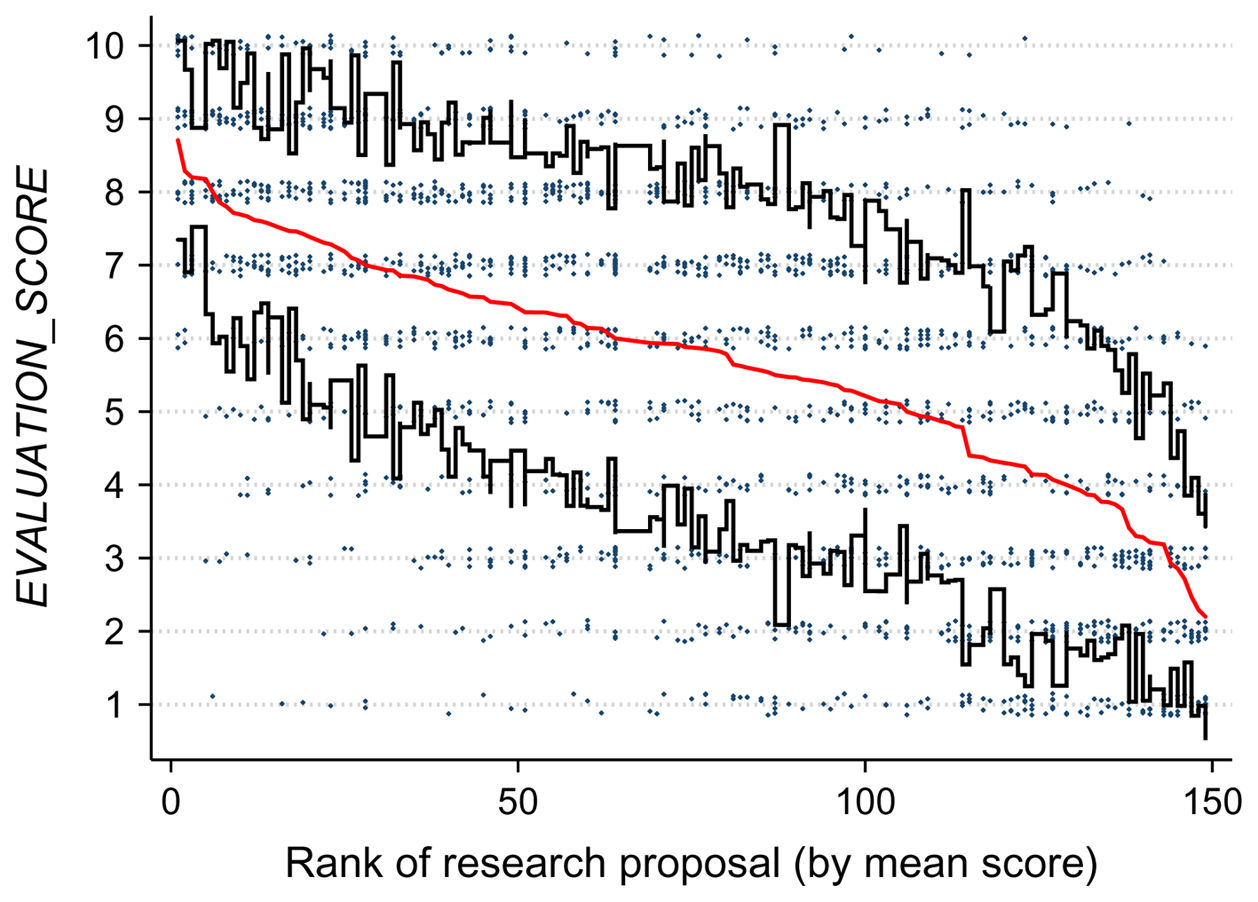
Evaluation Scores for Each Proposal, Ordered By Mean Scores (Mean and Plus/Minus One Standard Deviation Shown) *Note.* Individual integer scores are vertically randomly “jittered” to avoid overlap.

The main dependent variable, *EVALUATION_SCORE*, is the integer score from 1 to 10 (mean = 5.7; mode = 7; s.d. = 2.6) given by evaluators in response to the main scoring question. Figure 2 displays all scores assigned to each proposal. Proposals appear in descending order by average score, along the *x* axis. (The average score was the basis for conferring awards.) Figure 2 also presents the plus and minus of one standard deviationas a means of highlighting the consistently wide variation in evaluations across each proposal. The patterns are consistent with considerable noise in the evaluation process. For example, dummy variables for individual research proposals explain just 26% of variation in terms of the *R*^2^ statistic; dummy variables for individual evaluators explain 19% of variation in terms of the *R*^2^ statistic.

#### 3.4.2. Intellectual Distance Between Evaluators and Research Proposals

A first approach to measuring intellectual distance in our setup is simply to distinguish those evaluators who have previously published within the disease domain versus those who have not, as captured by the indicator variable *OUTSIDE_DOMAIN*. We also constructed a continuous measure of intellectual distance on the basis of keywords used to describe and categorize the content of research in the life sciences, collectively referred to as Medical Subject Heading (MeSH) terms. This is a controlled vocabulary used by the U.S. National Library of Medicine to index articles for PubMed. MeSH keywords are assigned not by authors but rather by professional science librarians trained specifically to perform this task. Use of this controlled vocabulary is intended to ensure global and consistent assignment of keywords across the life sciences (Coletti and Bleich 2001). We hired a professional librarian trained in standardized procedures for evaluating the content of research according to NIH National Library of Medicine (NLM) guidelines to code the proposals. We used the 2012 edition of the MeSH set, which contains 26,579 terms. On average, proposals in our sample were assigned 12.42 MeSH terms (s.d. = 5.42). This enabled us to represent each proposal as a vector of ones and zeroes, depending on relevant MeSH terms. We constructed analogous vectors to reflect evaluators’ backgrounds, with counts of numbers of papers referring to MeSH terms. Our continuous measure of intellectual distance is then simply the angular separation or cosine between the vectors for the proposal and the evaluator, expressed as a percentile, *EVALUATOR_DISTANCE*. The value of 1% reflects the closest and 100% the greatest intellectual distance. We refer to “evaluator” distance in naming this variable to emphasize that distance varies in relation to evaluator–proposal pairs. Formulating the variable as a percentile lead the distribution to be uniform and also eased interpretation; coefficients can be directly read as the effect of moving from the min (1st) to max (100th) percentile. (Alternative formulations of the variable produce similar results, as noted in the analysis.)

#### 3.4.3. Novel Departures of Proposals from Existing Research

Our measure of novelty is also based on the MeSH lexicon. MeSH keywords attributions are intended to capture key aspects of the research, including scientific approach, topic, methods, and other key issues. To develop a measure of novelty, we therefore simply looked for novelty in MeSH term combinations in relation to the existing literature. We compared the MeSH term combinations of a proposal with combinations that appear in the entire existing scientific literature, as reflected in the PubMed database.^4^ We examined all possible pairs of MeSH terms (i.e., for *N* terms there would be *N* (*N* − 1)/2 pairs) and determined what fraction of these pairs for a given proposal had not previously appeared in the accumulated literature. The variable is then expressed as the percentile, *PROPOSAL_NOVELTY*, with 1% being least and 100% most novel. We refer to “proposal” novelty in the naming of this variable to emphasize its relation to the proposal in relation to the broader stock of research rather than to any one evaluator. (Alternative formulations of the variable produce similar results, as noted in the analysis.)

#### 3.4.4. Other Variables

The analysis relies most heavily on the research design’s randomization and exploitation of multiple observations per proposal and per evaluator, with a series of dummy variables for evaluators and proposals providing controls. We also use a series of proposal covariates as a control vector (number of words, number of references cited, number of figures, presence of an introductory section that provides context in the proposal) where we cannot use proposal dummy variables. We discuss the relevance of these covariates in the analysis to follow.

## 4. Main Results

Here, we present our main results, estimating the relationship between evaluation scores and intellectual distance, and with proposal novelty. We report results in separate subsections, given that estimates of relationships with distance and novelty require different econometric approaches.

### 4.1. Intellectual Distance and Evaluation Scores

The evaluation of proposal *i* by evaluator *j* can be shaped by proposal covariates (*X_i_*) (e.g., underlying quality, type), evaluator covariates (*X_j_* ), and luck or noise, which we describe with a zero-mean error term (*ε_ij_* ). Regarding pairwise proposal–evaluators variation, our main focus here is on intellectual distance between evaluators and proposals (*EVALUATOR_DISTANCE*). (The design of our experiment controls for other pairwise factors, such as relationships among evaluators and researchers.) These variables relate to evaluation scores through some function *g*(−), *EVALUATION*_*SCORE_ij_* = *g*(*EVALUATOR*_*DISTANCE_ij_, X_i_, X_j_; ε_ij_* ). Our empirical models estimate this expression in a series of linearly separable specifications. Coefficients and robust standard error estimates are reported in Table 4.^5^

We begin with a most straightforward comparison between evaluation scores of those evaluators who have conducted research within the disease domain versus those who have not. As in Model 1, evaluation scores of those outside the disease domain are 0.37 points higher (s.e. = 0.12), on average. Given randomized assignment, adding proposal dummy variables, as in Model 2, does not change the estimated coefficient but reduces standard errors.^6^

Apart from discrete differences, we expect that continuous variation in intellectual distance will also shape evaluations. We therefore add our continuous measure, *EVALUATOR_DISTANCE*, to the model.^7^ As reported in Model 3, we again find a positive relationship with distance, the estimated coefficient on *EVAL-UATOR_DISTANCE* being 1.10 (s.e. = 0.19).

Importantly, using the continuous measure allows us to introduce evaluator dummy variables as controls. Thus, our preferred and most stringent specification includes dummy variables for both research proposals (*η*) and evaluators (*δ*) (with *OUTSIDE_ DOMAIN* dropping out of the model) as follows:

(1) $$\mathrm{EVALUETION}\_{\mathrm{SCORE}}_{ij}=\beta \cdot \mathrm{EVALUATOR}\_{\mathrm{DISTANCE}}_{ij}+\delta_{i}+\eta_{j}+\varepsilon_{ij},$$

where *ε* is a zero-mean error term. As reported in Model 4, this produces a slightly smaller, but statistically unchanged, coefficient on *EVALUATOR_ DISTANCE* (0.86, s.e. = 0.33).

Therefore, there is a large positive relationship between evaluation scores and intellectual distance. Given a random assignment of proposals to evaluators, the estimated relationship can be interpreted as a causal relationship. Therefore, not only do specialized experts provide more discerning evaluations but they also provide systematically lower—and more critical—evaluations. Having defined *EVAL-UATOR_DISTANCE* in terms of percentiles, we can interpret the coefficient as indicating a roughly one-point difference in score across the entire population, with varying intellectual distance, in addition to the earlier-reported 0.4 added points for those outside the research domain. This is a large effect in comparison with the standard deviation of evaluation scores, 2.6 (or a standard deviation of 1.7, if calculated after removing proposal and evaluator fixed effects).

### 4.2. Novel Departures from Existing Research and Evaluation Scores

We now examine the relationship between evaluation scores and novelty. Because this reintroduces a proposal covariate, *PROPOSAL_NOVELTY*, to the model, we can no longer exploit proposal dummy variables. Instead, we include a vector of precise proposal covariates, **X***_j_*, as control variables, as follows:

(2) $$\mathrm{EVALUETION}\_{\mathrm{SCORE}}_{ij}=\beta \cdot \mathrm{EVALUATOR}\_{\mathrm{DISTANCE}}_{ij}+\gamma \cdot \mathrm{PROPOSAL}\_{\mathrm{NOVELTY}}_{J}+\delta_{i}+\zeta \cdot X_{j}+\varepsilon_{ij},$$

where we continue to control for evaluator characteristics with dummy variables, *δ_i_*; *ζ* is the vector of parameters to be estimated on control variables. The error term is redefined accordingly. We control for differences in scores related to different specific fields and topics with the series of dummy variables of individual MeSH terms. We control for differences in quality with numbers of author publications and citations. We also control for a series of descriptive features of proposals (number of words, number of references cited, number of figures, presence of an introductory section that provides context in the proposal). Exploiting this control vector requires that we study just the subsample of 689 proposal–evaluator pairs for which we have these control variables (i.e., submissions from within the host university) rather than our full sample of 2,130 evaluator–proposal pairs. This leaves ample degrees of freedom, and the mean and variance of *EVALU-ATION_SCORE* are statistically the same in the sub-sample. Results are reported in Table 5.^8^

Model 1 regresses evaluation scores on *PRO-POSAL_NOVELTY*, together with evaluator dummy variables and the control vector of proposal covariates. The estimated coefficient on *PROPOSAL_ NOVELTY* is large and negative, at −2.67 (s.e. = 0.64). Most of the coefficients on proposal covariates are statistically significant. The exception is the number of words per proposal, which becomes insignificant when included with other proposal variables (but is positive and significant as other control variables are dropped). The control vector is highly effective at accounting for proposal characteristics; variation explained (unadjusted *R*^2^ = 0.428) is even almost the same as when proposal dummy variables were earlier included (unadjusted *R*^2^ = 0.475). Therefore, introducing the long list of controls leaves little room for lingering omitted variable bias—if only because there is little omitted variation.

If the model is indeed well controlled and there is little scope for unobserved proposal characteristics that spuriously account for the negative relationship with novelty, then introducing more controls should have no effect on estimates. To assess this point, Model 2 reestimates the model, adding controls for the number of author citations in the past seven years (in case the recency of citations plays a role), counts of publications in which the researcher appears as first author, and the maximum number of citations to any one of a researcher’s publications. As in Model 2, adding these controls has no effect on coefficient estimated on *PROPOSAL_NOVELTY*.^9^

Model 3 introduces *EVALUATOR_DISTANCE* into the model at the same time; the coefficient on *PROPOSAL_NOVELTY* is unchanged with this change. Further, the coefficient on *EVALUATOR_DISTANCE* is itself statistically unchanged from earlier estimates in Table 4 that used proposal fixed effects (rather than the control vector used here).^10^ This again affirms the effectiveness of our specification in isolating the relationship of interest. (As discussed in §5.1, there is no interaction between distance and novelty.) Therefore, the all-else-being-equal relationship between evaluation score and novelty is negative.

**Figure 3:**
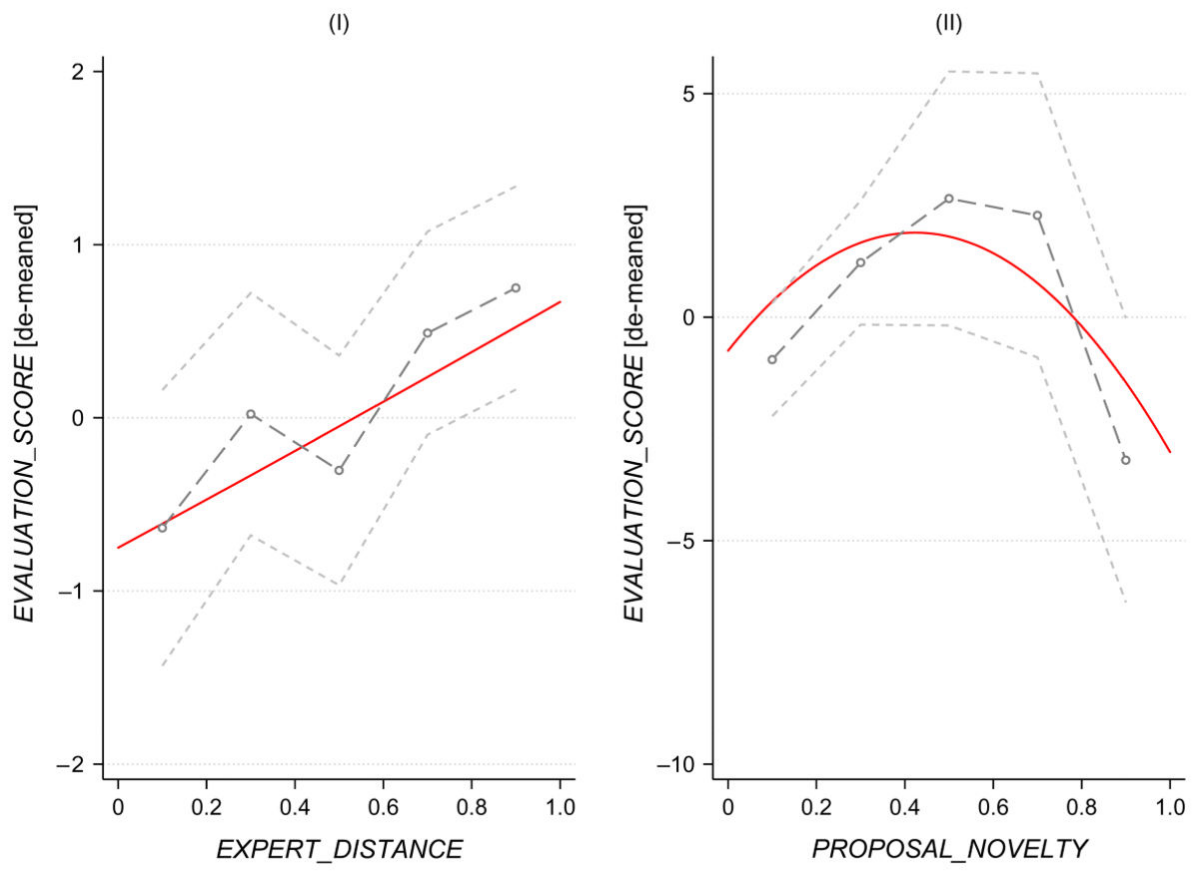
Flexible, Nonlinear Specification (Second-Order Polynomial and Quintile Means) *Notes.* Shown are 90% confidence intervals. See §5.2 for discussion of specifications.

Having established the meaningfulness and stability of our model specification, we move to investigating whether the relationships of interest are nonlinear. In Figure 3, we present results in which we allow for nonlinear relationships between evaluation scores and our measures of intellectual distance and novelty in two different specifications. The first approach is to simply add quadratic terms for both distance and novelty to the model (while continuing to control for evaluator fixed effects and a full complement of proposal covariates as controls). The second approach is to include a series of dummies for different levels (five quintiles) of both intellectual distance and novelty variables, estimating them in the same model. (Estimating the relationships with distance and novelty simultaneously or in separate models leads to similar patterns.) We present the estimates related to distance and novelty across these two models in the two panels of Figure 3. They each produce similar results. We find that the relationship with intellectual distance is in fact linear and increasing, with no evidence of nonlinearities. Both models also indicate that the negative relationship between evaluation scores and novelty is largely driven by the most novel proposals. These are proposals in the fifth quintile of novelty, which might be understood as the right tail of novelty. The overall relationship is nonmonotonic, because at low levels of novelty there is an increase of scores with increases in novelty.

## 5. Evaluation of Alternative Explanations

Here, we interpret results in light of theoretical perspectives described in §2.2 (summarized in Table 1). We find that it is only the third of the perspectives we consider here—a bounded rationality characterization of the evaluation process—that is wholly consistent with patterns observed here. Thus, the first two subsections primarily deal with ruling-out possible explanations, and it is the third that finally rules in an explanation.

### 5.1. Agency Problems and Private Interests

Here, we consider the possibility that agency problems lead some evaluators to bias their evaluations upward or downward, depending on how they perceive that “close” research proposals will influence their own careers and private interests (§2.2.1). A series of patterns in the data run counter to this explanation.

#### 5.1.1. Mean Responses and Bias

One possible interpretation of agency problems is that both low intellectual distance and low novelty can be regarded as close research. Therefore, whatever the general directional response to close research, we would expect the same direction of response in relation to both low novelty and low distance. However, in earlier analysis we measured that distance and novelty relate to scores with opposite signs.

Another possible interpretation of agency problems is that research that is at low intellectual distance is close, but it is only close research that is at the same time highly novel that might present a competitive threat to evaluators. However, we find no significant interaction between novelty and distance in explaining evaluation scores (see Table 6, Model 1).

In addition, if private interests were to play some sort of systematic role, we would expect to see a heightened response (of some sort) to research that is *especially* close, perhaps resulting in a step function response on scores or at least some sort of nonlinear effect or impact on variance of scores. However, the relationship between scores and intellectual distance is linear with no signs of outsized response or even greater variance in the case of close research.

#### 5.1.2. Heterogeneous Responses Across the Distribution of Evaluators

To investigate the possibility that certain evaluators are perhaps more susceptible than others to agency problems and bias and that this might result in heterogeneous responses across evaluators, we reestimate the model described in expression (3), allowing the coefficient on *EVALUATOR_DISTANCE* to be heterogeneous across evaluators $\beta_{i}\sim N(\bar{\beta },\sigma_{\beta }^{2})$.

**Figure 4:**
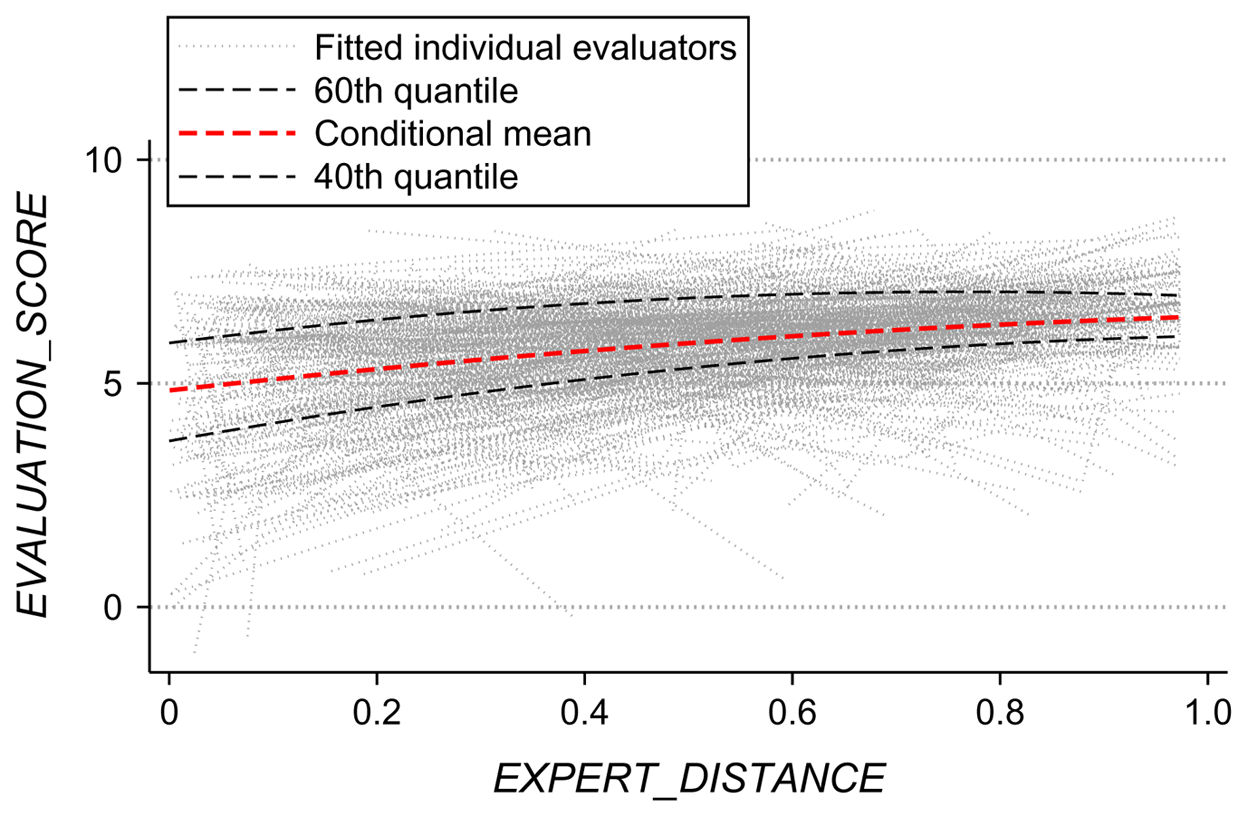
Fitted Linear Relationships for Individual Evaluators *Note.* Quantile and mean fitted lines are also shown to provide additional perspective on the distribution of data; each is regressed as a second-order polynomial.

Estimating this random coefficient specification, we find that the mean coefficient on our intellectual distance variable, *β̄*, is 1.48 (s.e. = 0.41), and we estimate that the standard deviation of this coefficient across the population of evaluators, *σ_β_*, is 0.61 (s.e. = 0.32). The relative size of the positive mean to standard deviation indicates that the response to intellectual distance is overwhelmingly mostly positive across the population. To underline this point, Figure 4 plots fitted individual linear relationship estimates for each evaluator across the multiple proposals they each evaluated, demonstrating the consistency of responses across evaluators.

#### 5.1.3. Interactions and Evaluator Types

As still another test for agency problems, we examine whether effects of *EVALUATOR_DISTANCE* somehow systematically vary with factors plausibly linked to strength of self-interest, strategic orientation, and susceptibility to agency problems. As reported in Table 6, we test for possible interactions with novelty (Model 1), evaluator seniority (Model 2), years since graduating (Model 3), and gender (Model 4), as well as all interaction terms at once (Model 5). We find no significant interactions.

### 5.2. Uncertainty, Risk, and Decision Theory Perspectives

Here, we consider the possibility that the evaluation process is analogous to a statistical decision-making problem, whereby greater distance and uncertainty creates noisier “signals” of the unobserved true quality of proposals (see §2.2.2).

It is also difficult to reconcile this perspective with the data, beginning with the most basic mean associations. For example, on the one hand, this perspective predicts that expected true mean assessment of quality should be invariant to distance and uncertainty. But evaluator scores systematically varied with both distance and novelty. On the other hand, it is possible that evaluations were risk-discounted relative to the expected mean quality, as uncertainty and distance increase. However, instead of a negative relationship with distance, we see a positive one. Only the relationship with novelty is negative.

#### 5.2.1. Dispersion and Variance

This perspective also suggests the possibility of greater uncertainty leading to wider variance and dispersion of evaluations with varying distance or novelty. Simple descriptive statistics provide no indication of differences in variance at low and high levels of either intellectual distance or novelty. For example, the standard deviations of evaluation scores for fifth quintiles of either distance or novelty are no different from the standard deviation for lower quintiles. To investigate this possibility, here we reestimate the earlier model but allow the model error term to vary with novelty and distance, redefining the error term as *m_ij_* · *ε_ij_*, where multiplier *m* is allowed to vary with key explanatory measures: *m_ij_* = 1 + *β^ε^* · *EXPERT*_*DISTANCE* + *γ^ε^* · *PROPOSAL*_*NOVELTY_j_*. We simultaneously estimate conditional mean and error model coefficients via maximum likelihood. Coefficients in the conditional mean model are statistically unchanged in this specification, and estimated coefficients in the error term multiplier expression are statistically indistinguishable from zero (*β^ε^* = −0.21, s.e. = 0.17; *γ^ε^* = −0.19, s.e. = 0.16). (Reestimating the multiplier model with quadratic terms or any subset of the variables, one at a time, produces the same zero result.) Therefore, we find no evidence of changing variance and dispersion with either distance or novelty.

### 5.3. Bounded Rationality and Expert Cognition Perspectives

Here, we consider the possibility that uncertainty is sufficiently high in evaluations where heuristic and behavioral decision making play a prominent role—a bounded rationality perspective (see §2.2.3).

As regards intellectual distance, this perspective suggests that those with most relevant knowledge, closest experts, will better discern informational cues, sample from a wider array of information, and make better sense of these cues. Following this perspective, the finding of a positive relationship between evaluations and intellectual distance (more negative evaluations by closest experts) is consistent with experts being more critical—applying more extensive tests, uncovering more errors, problems, and limitations.

To seek additional evidence related to bounded rationality and intellectual distance, we compared rank ordering based on the 15 randomly assigned evaluators with rank ordering based on scores given by intellectually closest evaluators (“experts”) for each proposal from 150 proposals.^11^ On average, rank order is different by a staggering 31.8 positions (s.d. = 26.0).^12^ To look for evidence as to whether experts provide more discerning evaluations than do less expert groups, we examine whether reducing the idiosyncratic noise of expert evaluations (taking out individual fixed effects and correcting for varying distance) leads expert rank ordering and less expert group average rank ordering to become more or less similar.^13^ We find that taking away noise from expert rankings leads them to become more different from rankings of less expert group averages—but only for high-quality proposals. These patterns are consistent with expert evaluations being more discerning in relation to the subtle differences separating high-quality proposals.

As regards novelty, the bounded rationality perspective suggests that established knowledge and mental models are “brittle,” and this leads to systematic errors in judging new ideas (see §2.2.3). This is consistent with our finding of a negative relationship between evaluation scores and novelty. This is also perhaps consistent with the negative relationship being largely driven by the most novel proposals (see Figure 3). For example, some minimal amount of novelty is necessary in making a research contribution. However, it is perhaps just largest novel departures that are most likely to be misconstrued and discounted.

Each of the other patterns documented in this and previous sections are themselves also reconcilable with the bounded rationality perspective. For example, the “smooth” and gradually changing relationships documented in Figure 4 are consistent with gradual changes in cognition and behaviors with incrementally varying uncertainty. The similar behavior across the wide cross section of evaluators (see Figure 4) and absence of distinct effects across different groups (see Table 6) is consistent with the universal effects of bounded rationality. The invariance of dispersion in evaluations with varying levels of uncertainty—whether measured in terms of distance or novelty (see §5.2)—is consistent with common behavioral and heuristic responses to uncertainty.

## 6. Summary and Conclusions

This paper reported the results of an experiment designed to evaluate how evaluation scores of scientific research proposals are related to intellectual distance (between evaluator knowledge and the knowledge content of research proposals) and novelty (of research proposals in relation to the body of accumulated research). We conducted our field experiment as part of a regular research grant proposal process involving a group of world-class medical researchers. We worked closely with grant administrators to alter and manipulate features of a grant proposal process to implement a controlled research design. We focused on effects of relative positions in “knowledge space” (intellectual distance and novelty), striving to isolate these effects from the many other factors plausibly influencing evaluations. Important in this regard, we implemented a triple-blinding procedure while having individual evaluators (working in isolation) evaluate single-authored research proposals that followed a standard format. Randomization of assignment allowed us to estimate causal effects of intellectual distance (between evaluator–proposal pairs) on evaluation scores, holding proposal and evaluator characteristics constant. It is, of course, not possible to experimentally vary the novelty of a proposal entirely independently of its other characteristics. Therefore, we implemented a best feasible approach to estimate the relationship between novelty and evaluation scores, all else being equal. Given limitations in observing true quality and potential of research initiatives—even ex post—a key feature of our research design is to derive inferences without relying on observing true quality.

### 6.1. Results

We found that evaluators gave systematically lower scores to research proposals that were closer to their own areas of expertise. Within the range of variation observed here, the effect of intellectual distance on evaluation scores was large—a 1-point or more difference on a 10-point scale. These effects are observed across a wide cross section of evaluators. By contrast, we found no evidence of changing variance (deviations from mean model predictions) with varying intellectual distance. Therefore, closer experts were systematically more critical in the sense of assigning lower scores.

Our second main finding is that more novel proposals are associated with lower evaluations. The size of the relationship is large and comparable in magnitude, in these data, to the earlier effect of intellectual distance on evaluation scores. It is proposals with particularly high levels of novelty that account for this result. (For low levels of proposal novelty, evaluation scores were increasing with incrementally greater levels of novelty.) We found no evidence of changing levels of variance in scores at different novelty levels. A series of alternative specifications and diagnostic tests all but rule out the possibility that unobserved proposal characteristics somehow account for the observed patterns.

### 6.2. Interpretation

We considered a range of possible explanations for the patterns. Only theories emphasizing the bounded rationality of evaluators (Kahneman et al. 1982, Johnson et al. 1982, Camerer and Johnson 1991) provide explanations for all observed patterns, on their own.

In relation to intellectual distance, bounded rationality characterizations suggest that closer experts “see” or “sample” more informational cues than do nonexperts. (There is no reason to expect that added informational cues seen by experts should necessarily be sampled equally from both merits and demerits of a proposal.) The pattern of lower scores provided by most expert evaluators is consistent with experts more readily discerning added demerits, problems, and limitations of research proposals rather than hidden demerits, in relation to what is perceived by less expert evaluators. Also consistent with this interpretation, counterfactual simulations comparing experts with wider groups suggested expert judgment was especially more discerning when judging higher-quality proposals (where differences in quality are presumably more subtle).

In relation to novelty, a bounded rationality characterization suggests that experts extrapolating beyond the knowledge frontier to comprehend novel proposals are prone to systematic errors, misconstruing novel work. This implies that rather than receiving unbiased assessments (with zero-mean errors), novel proposals are discounted relative to their true merit, quality, and potential.

Other theories failed to reconcile with all or some of the patterns documented here. For example, the patterns are inconsistent with evaluators shading their scores in relation to private interests. The patterns are also inconsistent with a statistical decision theory perspective in which evaluations simply become more “noisy” with greater distance and uncertainty.

The negative relationship between evaluation scores and proposal novelty is, however, consistent with possible discounting on the basis of uncertainty and ambiguity (Fox and Tversky 1995). But this leaves the question of why greater uncertainty from greater intellectual distance is not then also discounted. It is plausible that the ambiguity associated with novelty plays some sort of special role, whereas uncertainty associated with distance does not, but then ambiguity only exists in a context of bounded rationality, complementing the earlier bounded rationality interpretation of patterns.

Our analysis ruled out the possibility that unobserved proposal characteristics that are unrelated to novelty somehow accounted for novel proposals receiving lower evaluations. Nonetheless, on a much subtler point, it remains possible that novelty per se is unavoidably and inextricably linked to lower expected mean outcomes (cf. Fleming 2001). However, if evaluations were to, in fact, reflect true quality, it remains then a question why we do not also see greater variance of evaluations associated with novel proposals to reflect greater true variance of these proposals (Fleming 2001).

As regards generalizability of findings, we should emphasize that evaluators in these data were each world-class medical researchers drawn from both inside and outside the disease area (endocrine-related disease). This implies a span of intellectual distance that is perhaps closer to that within distinct subfields of natural sciences, engineering, or social sciences—rather than to larger differences across distinct fields. Just as we did not study especially intellectually distant evaluators, we also did not study differences between experts and lay people here.

### 6.3. Contributions and Relationships to Literature

Our work relates to the evaluation of frontier innovative projects. However, it most specifically contributes to several decades of research on scientific evaluations. To date, the bulk of this research has been carried out in fields of life sciences, medical research, and science policy (see, e.g., Cole et al. 1981, Chubin and Hackett 1990, Lee et al. 2013) with yet limited attention from social scientists, economists, and management scholars. Within this literature, our paper adds to the few studies attempting to make causal empirical inference (e.g., McNutt et al. 1990, van Rooyen et al. 1999, Li 2015). We believe that pursuit of careful causal inference (along with careful distinctions between underlying mechanisms) is especially important both because of the potentially staggering consequences of resource (mis)allocation in innovation and science and because many of the theories and claims in this literature are both complex and controversial.

Certainly, questions of “distance” between researchers and evaluators have been considered in research on scientific evaluations, but the focus thus far has been on distance between evaluators and researchers in terms of factors such as race, gender, and social and professional networks. Our work departs by instead considering relative positions and distance in knowledge space between evaluators and the proposals (rather than the researchers) they evaluate. In this regard, our study is closest to Li’s (2015) study of committee grant evaluations at the NIH. She finds that proposals citing committee members are more likely to win a grant. Further, grant decisions on proposals citing committee members are more closely correlated with a proxy for research quality (a citation-based measure) than are proposals that do not cite committee members. Li interprets these patterns as committees generally not only favoring researchers who cite them but also being more familiar with those researchers. Especially relevant here, her finding that citing proposals having a *greater* probability of being granted is directionally *opposite* to our finding that close intellectual proximity causes *lower* evaluations. This might relate to a number of institutional details of the NIH context that differ from our artificially manipulated and controlled environment, including researcher identity not being blinded, the evaluation process being conducted via open committee discussions, and potentially differing levels of variation observed in intellectual distance. It is also possible that the sorts of researchers with a high hazard of citing NIH committee members differ from those with a low hazard of doing so. Li’s study thus complements our own in highlighting the importance of additional research in seeking to better comprehend how these added issues and mechanisms drive outcomes in evaluation.

Our research also relates to (and was inspired) the observation that research, innovation, and technical advance tend to advance in an incremental fashion within defined *paradigms*, *knowledge trajectories*, *research pathways*, or *dominant designs*—some basic approach to solving the problem—that is then incrementally refined through a continuous series of cumulative, incremental advances (e.g., Kuhn 1962, Dosi 1982, Sahal 1985, Romer 1990, Gibbons et al. 1994, Murray and O’Mahony 2007, Furman and Stern 2011, Williams 2013, Boudreau and Lakhani 2015). This overriding tendency toward within-paradigm, incremental advance rather than more novel and exploratory innovations might be explained by any number of mechanisms, such as the strategic incentives and organization of innovators (e.g., Kuhn 1962, Utterback and Abernathy 1975, March 1991, Manso 2011). The current paper raises another possibility: even when novel projects are proposed, they are met with resistance from relevant gatekeepers and purse holders.

### 6.4. Implications for the Evaluation of Frontier Projects

Regarding intellectual distance, the earlier analysis suggests that closest expert evaluations can offer more discerning evaluations than can more distant evaluators. A challenge, however, in implementing expert reviews is that different experts might be needed to evaluate different proposals, introducing evaluator-specific idiosyncratic noise. One possible remedy is to algorithmically correct for individual fixed effects and variation in distance (as we did earlier). Where data are not available, perhaps senior evaluators can more informally make judgments to virtually correct for evaluator differences. The results highlight the limits of aggregating the views of more distant evaluators who individually offer more superficial evaluations. Lesser experts simply cannot “see” what experts can see. It is not clear whether this is a problem that can be solved by averaging and aggregation.

The challenges related to novelty are greater still. Any discounting of novel proposals relative to true quality implies underinvestment in novel proposals. No amount of aggregation and averaging, blinding, or other conventional policies can address this problem. Plausible avenues to address this problem include priming and coaching evaluators to create greater understanding and awareness of resource allocation goals and their own cognitive limits. As our analysis demonstrates, it is also possible to supplement evaluations with statistics providing objective measures of the degree of novelty of a given proposal. Programs geared to providing researchers with less stringent constraints in allocating resources might also play a role in fostering novel innovation (e.g., Manso 2011). However, this presumes that innovators themselves may be better able to judge the merits of novel projects than will independent evaluators—something not addressed in this study.

Our findings suggest still other reasons for under-investment in novelty that do not depend on whether evaluations discount novel proposals relative to true expected outcomes. Consider that innovators might, in principle, wish to trade off lower expected mean innovation outcomes for greater ex post variance of outcomes, and possible upside risk (Fleming 2001). However, here we found no evidence whatsoever of greater variance in the ex ante evaluations of novel proposals. This raises the question of whether—in the context of bounded rationality, uncertainty, and ambiguity—evaluators can even *perceive* the high variance potential of novel proposals ex ante (let alone implement the trade-off).

More broadly, these points regarding bounded rationality in the evaluation of novel proposals raise the question of what evaluators (and innovators) can hope to know, plan for, and anticipate as they pursue novel exploration. How utterly uncertain is such experimentation ex ante? Recent studies of ex post patterns of outcomes with novel innovations suggest that it is productive for foresighted innovators and evaluators not only to promote novel proposals but to promote quite particular kinds of novel proposals (e.g., Uzzi et al. 2013, Kaplan and Vakili 2015). And yet it is far from clear that evaluators (or innovators) can be all that foresighted to steer their innovation in these particular directions. These are questions for future research.

## Figures and Tables

**Table 1:** Alternative Theoretical Mechanisms Possibly Relating Intellectual Distance to Evaluations

Theoretical perspective	Mechanism	Predicted relationship of intellectual distance to mean evaluation	Predicted relationship of novelty to mean evaluation	Predicted relationship with variance of evaluations
2.2.1 Uncertainty, risk, and decision theory	•Distance, uncertainty, and dispersion	(−)		(+)
	•Discounting and risk adjustments	(−)		
	•Discounting and ambiguity aversion		(−)	
2.2.2 Agency problems and private interests	•Promotion of one’s own work or “schools of thought” or “protecting” existing approaches	(−)		
	•Discounting competing research	(+)		
2.2.3 Bounded rationality and expert cognition	•More discerning and extensive assessments and tests by experts	(+)		
	•Systematic errors when using existing models to extrapolate to new domains		(−)	

**Table 2:** Definitions of Main Variables

Variable	Description
(1) *EVALUATION_SCORE*	Main integer score from 1 to 10 given by an evaluator to a research proposal
(2) *OUTSIDE_DOMAIN*	Indicator switched to 1 for those evaluators who have not previously published on endocrine-related disease
(3) *EVALUATOR_DISTANCE*	With evaluators and research proposals each represented as vectors of (MeSH term) keywords, this variable is the cosine of the angle between the vectors, expressed as a percentile (1% to 100%)
(4) *PROPOSAL_NOVELTY*	Of all the keywords (MeSH term) used to describe a research proposal, the share of these terms not yet observed in prior published research, expressed as a percentile (1% to 100%)
(5) *WORDS*	Total number of words in main text of each proposal
(6) *NUM_REFS*	Total number of references listed in each proposal
(7) *NUM_FIGS*	Total number of figures shown in each proposal
(8) *INTRO_SECTION*	Indicator switched to 1 for those proposals which begin with an overview or introduction section
(9) *AUTHOR_PUBS*	Count of all publications of the researcher submitting the research proposal
(10) *AUTHOR_CITES*	Count of all citations of prior publications of the researcher submitting the research proposal

**Table 3:** Means, Standard Deviations, and Correlations of Main Variables

Variable	Mean	s.d.	(1)	(2)	(3)	(4)	(5)	(6)	(7)	(8)	(9)
(1) *EVALUATION_SCORE*	5.69	2.58									
(2) *OUTSIDE_DOMAIN*	0.65	0.48	0.03								
(3) *EVALUATOR_DISTANCE*	0.50	0.29	0.15	0.00							
(4) *PROPOSAL_NOVELTY*	0.50	0.29	−0.03	−0.01	0.10						
(5) *WORDS*	1,366	2,489	0.02	0.01	0.03	0.12					
(6) *NUM_REFS*	5.61	9.76	0.11	0.00	0.08	0.02	0.40				
(7) *NUM_FIGS*	0.28	0.84	0.04	−0.01	0.05	0.01	0.35	0.55			
(8) *INTRO_SECTION*	0.21	0.41	0.05	0.01	−0.06	0.04	0.16	0.07	0.07		
(9) *AUTHOR_PUBS*	9.13	24.01	0.03	−0.01	0.12	−0.07	0.00	0.00	−0.07	0.23	
(10) *AUTHOR_CITES*	99	521	0.07	−0.01	0.16	0.08	0.05	−0.03	−0.06	0.31	0.90

**Table 4:** Estimated Relationship Between Evaluations (*EVALUATION_SCORE*) and Intellectual Distance Between Evaluators and Research Proposals (*EVALUATOR_DISTANCE*)

	Dependent variable: *EVALUATION_SCORE*			
	1	2	3	4
	Outside of disease domain	Control evaluator chars.	Continuous measure of distance	Control evaluator and proposal chars.
*OUTSIDE*_ *DOMAIN*	0.37*** (0.12)	0.37*** (0.10)	0.36*** (0.10)	
*EVALUATOR*_ *DISTANCE*			1.10*** (0.19)	0.86*** (0.33)
Research proposal dummies		Y	Y	Y
Evaluator dummies				Y
Adj. *R*^2^	0.004	0.263	0.275	0.475

*Note*. Heteroskedasticity-autocorrelation robust standard errors are reported; number of observations = 2,130 research proposal–evaluator pairs.

*, **, and *** indicate statistical significance at the 10%, 5%, and 1% levels, respectively.

**Table 5:** Estimated Relationships Between Evaluations and Proposal Novelty

	Dependent variable: *EVALUATION_SCORE*		
	Model 1	Model 2	Model 3
	Evaluator dummies and proposal control vector	Extended proposal controls	Distance and novelty
*PROPOSAL_NOVELTY*	−2.67*** (0.64)	−3.10*** (0.89)	−2.80*** (0.64)
*EVALUATOR_DISTANCE*			1.48** (0.59)
	Evaluator controls		
Evaluator dummies	Y	Y	Y
	Research proposal controls		
Researcher quality			
*AUTHOR_PUBS*	−0.15*** (0.03)	−0.14*** (0.03)	−0.15*** (0.03)
*AUTHOR_CITES*	0.005*** (0.00)	−0.02 (0.01)	0.006*** (0.00)
Extended set of controls^a^		Y	
Research type			
Keyword (topic) dummies	Y	Y	Y
Number of keywords	Y	Y	Y
Proposal characteristics			
*WORDS*	0.00 (0.00)	0.00 (0.00)	0.00 (0.00)
*NUM_REFS*	0.10*** (0.04)	0.04 (0.04)	0.10*** (0.04)
*NUM_FIGS*	−1.12** (0.45)	−1.14** (0.57)	−1.18** (0.45)
*INTRO_SECTION*	1.85*** (0.41)	1.35*** (0.50)	1.94*** (0.41)
Adj. *R*^2^	0.423	0.459	0.428

*Note*. Heteroskedasticity-autocorrelation robust standard errors are reported; number of observations = 689 proposal–evaluator pairs and pertain only to submitting researchers from within the host university.

a Number of author citations in the past seven years, counts of publications in which the researcher appears as first author, maximum number of citations to any one of a researcher’s publications.

*, **, and *** indicate statistical significance at the 10%, 5%, and 1% levels, respectively.

**Table 6:** Interactions Between Evaluator Distance and Factors Plausibly Influencing Incentives and Behaviors

	Dependent variable: *EVALUATION_SCORE*				
	Model 1	Model 2	Model 3	Model 4	Model 5
*EVALUATOR_DISTANCE*	1.79** (0.78)	1.88*** (0.72)	1.64*** (0.58)	1.41** (0.64)	2.09** (0.98)
*DISTANCE* × *NOVELTY*	−0.38 (1.06)				−0.55 (1.04)
*DISTANCE* × *SENIOR*		−0.49 (0.74)			−0.59 (0.74)
*DISTANCE* × *YEARS SINCE GRAD*			0.00 (0.00)		0.00 (0.00)
*DISTANCE* × *FEMALE*				0.35 (0.75)	0.44 (0.74)
Evaluator dummies	Y	Y	Y	Y	Y
Research proposal dummies	Y	Y	Y	Y	Y
Adj. *R*^2^	0.482	0.482	0.482	0.482	0.480

*Note*. Heteroskedasticity-autocorrelation robust standard errors are reported; number of observations = 689 proposal–evaluator pairs and pertain only to submitting researchers from within the host university.

*, **, and *** indicate statistical significance at the 10%, 5%, and 1% levels, respectively.

## Footnotes

1 See Li (2015) for an approach using a form of proxy measure for true quality.

2 Sources of bias considered include social category, status and prestige, sex, nationality, language, and relationships between evaluators and researchers.

3 We tested for and found no evidence of statistical differences across the blocks.

4 PubMed includes approximately 185 million MeSH term combinations (from a body of 26,579 unique terms) assigned to some 21 million articles published between 1855 and 2010.

5 Alternative specifications allowing for truncation or for the nonnegative integer nature of the dependent variable do not alter the results.

6 Of those doing research outside of the disease domain, roughly half had and half did not have a coauthor publishing within the domain. We find no differences in evaluations between these subgroups.

7 Alternative measures, such as the simple cosine (not expressed as a percentile), Euclidean distance, and simple counts of overlapping areas, produce similar results.

8 The results do not depend on whether novelty is measured as a share or absolute number of new keyword pairs, triplets, or quadruplets; whether the variable measures departures from the last 10 years or the entire history of the literature on the PubMed database; or whether the model controls for the absolute numbers of keywords.

9 As additional assessments of the possibility that omitted variable bias is the reason for the negative relationship with novelty, we also considered effects of removing controls. When examining all possible combinations of control variables (including the main control vector and in the added controls introduced in Model 2), we find that progressively adding more controls to the model generally produces *more* negative estimates, not *less*. For example, dropping control variables altogether produces a far less negative coefficient (−0.25; s.e. = 0.20).

10 This is also the case despite these latter estimates being based on just a subset of authors for whom we observe control variables.

11 Mean *EVALUATOR_DISTANCE* is 0.13 (i.e., 13th percentile) for closest experts and 0.50 for groups.

12 Even among the top 25 ranks, the mean absolute change in rank positions when pursuing this alternative policy of evaluation by lone experts would have been 23.8 positions (s.d. = 27.0).

13 The rationale is that if reducing noise makes expert evaluations more similar to group averaged rank orderings, then we can presume that it is the noise rather than signal of expert evaluations that leads to the divergence. By contrast, if reducing noise only increases the divergence in ranking, then we can presume that it is the random noise that makes expert evaluations similar to the group averages (and that by reducing noise we are isolating a more meaningful signal).
